# Hybrid Deep Learning Framework for Sleep Quality Prediction: Integrating Metaheuristic Optimization and Statistical Features

**DOI:** 10.1002/brb3.71360

**Published:** 2026-04-14

**Authors:** Ayodele Lasisi, Nitasha Rathore, Lalita Gupta, Kanika Thakur, Shrikant Burje, Madhumathi Ramasamy, Sandeep Bhad, Anurag Sinha, Amar Jeet, Shakti Singh, Quadri Noorulhasan Naveed, Raman Kumar, Syed Abid Hussain

**Affiliations:** ^1^ Department of Computer Science College of Computer Science King Khalid University Abha Saudi Arabia; ^2^ Department of Computer Science and Engineering Bharati Vidyapeeth's College of Engineering New Delhi India; ^3^ Electronics and Communication Engineering Department Maulana Azad National Institute of Technology (MANIT) Bhopal India; ^4^ Department of Computer Science and Engineering Galgotias University NOIDA UP India; ^5^ Department of Electronics and Telecommunication RSR Rungta College of Engineering & Technology, Bhilai,Chhattisgarh, India Bhilai Chhattisgarh India; ^6^ Department of Computer Science and Engineering Sri Ramakrishna Engineering College Coimbatore India; ^7^ Department of Electronics and Telecommunication Engg.School of Engineering and Technology Rungta International Skill University Bhilai.CG, India. Bhilai Chhattisgarh India; ^8^ Department of Computer Applications, Sri Satya Sai University of Technology and Medical Sciences (SSSUTMS), Sehore, Madhya Pradesh, IndiaResearch Scholar, Department of Information Technology, Guru Ghasidas Vishwavidyalaya, Bilaspur, Chhattisgarh India; ^9^ Department of Mathematics and Computing BIT Mesra Ranchi India; ^10^ Lovely Professional University Phagwara Punjab India; ^11^ Department of Mechanical and Production Engineering Guru Nanak Dev Engineering College Ludhiana Punjab India; ^12^ Jadara Research Center Jadara University Irbid Jordan; ^13^ Department of Mechanical Engineering Graphic Era (Deemed to Be University) Dehradun India; ^14^ Department of Computer Science and Engineering Bakhtar University,University Centre for Research & Development, Chandigarh University, Gharuan, Mohali, Punjab India

**Keywords:** actigraphy data, digital biomarkers, feature selection, hybrid deep learning, long short‐term memory, sleep quality prediction, metaheuristic optimization, support vector machine

## Abstract

Assessing sleep quality is essential to preserving optimum health and well‐being, with consequences ranging from preventing chronic diseases to improving cognitive function. This paper introduces a sophisticated hybrid deep learning architecture that far outperforms current techniques for actigraphy data‐based sleep quality prediction. Our method uses two metaheuristic optimization approaches (genetic algorithms and particle swarm optimization (PSO)) for feature selection and combines statistical characteristics with complex features retrieved using long short‐term memory (LSTM) networks. support vector machines (SVMs) are then used to classify the optimized feature set. Our model outperforms baseline LSTM and other cutting‐edge methods when tested on the benchmark MESA Actigraphy dataset. It achieves remarkable accuracy (84.64% for weekly sleep quality and 68.99% for sleep consistency), F1‐scores (0.847 and 0.69, respectively), and AUC values (0.909 and 0.839, respectively). Furthermore, we close a significant gap in black‐box deep learning techniques by introducing a unique feature significance analysis that gives the model's predictions interpretability. Our results emphasize the potential of hybrid deep learning frameworks for individualized sleep health management and early diagnosis of sleep disorders by demonstrating the efficacy of integrating metaheuristic optimization with multimodal data in sleep quality prediction.

## Graphical abstract

This graphical abstract presents a hybrid deep learning framework for objective sleep quality prediction using wearable actigraphy data. Raw activity signals are preprocessed through normalization, resampling, and sliding‐window segmentation. Temporal sleep patterns are automatically learned using a long short‐term memory (LSTM) network, while complementary statistical features capture global activity characteristics. A dual metaheuristic optimization strategy based on a genetic algorithm and particle swarm optimization (PSO) selects the most informative features. The optimized feature set is classified using a support vector machine (SVM), enabling accurate, interpretable, and non‐intrusive assessment of weekly sleep quality and sleep consistency.

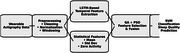

## 1 Introduction

A key component of human health, sleep quality has a significant impact on mental clarity, emotional control, physical recuperation, and general well‐being (Acebo et al. 2003). About one‐third of people suffer from sleep‐related problems, according to the World Health Organization, which has declared sleep disorders to be a worldwide health crisis (Andreotti et al. 2018). Severe psychological hazards, such as sadness, anxiety, reduced cognitive performance, and even suicidal thoughts, have been connected to chronic sleep loss caused by either inadequate length or poor quality (Arora et al., 2023; Arora et al., 2020). On the other hand, getting enough good sleep at the right times is crucial for maintaining mental health, memory consolidation, and brain function (Benca et al. 2019; Berry et al. 2012).

Traditionally, polysomnography (PSG), the gold standard for monitoring several physiological indicators during sleep, has been used to measure the quality of sleep (Breiman 2001). However, because of the intrusive nature of the technology, PSG is costly, necessitates specialist facilities, and may interfere with natural sleep patterns (Buysse et al. 1989). Self‐reported surveys like the Pittsburgh Sleep Quality Index (PSQI) are alternative approaches, although they are prone to subjective interpretation and recall bias (Carskadon 2021). These constraints have spurred the creation of more approachable, impartial, and scalable methods for evaluating sleep quality.

The Internet of Medical Things (IoMT) and wearable technology have recently proliferated, revolutionizing sleep monitoring by allowing continuous, non‐invasive data collection in natural settings (Cellini et al. 2020; Cole et al. 1992). Particularly, actigraphy devices have become well‐known as useful instruments for tracking sleep‐wake cycles via movement detection (Collop et al. 2011). These gadgets gather useful “digital biomarkers” that can reveal information about the structure and quality of sleep (Cortes and Vapnik 1995). However, because sleep patterns are complicated and vary widely across individuals, accurately interpreting actigraphy data and identifying important patterns continues to be a major difficulty (Diekelmann and Born 2014).

Deep learning techniques have become effective tools for deciphering complicated physiological data because they can automatically identify pertinent features without requiring a lot of manual feature engineering (Fong et al. 2018; Graves 2013). A kind of recurrent neural network called a long short‐term memory (LSTM) network has demonstrated significant promise in the analysis of sequential data, including actigraphy recordings (Hochreiter and Schmidhuber 1997; Holland 1975). However, deep learning models frequently operate as “black boxes,” being difficult to understand and occasionally failing to capture crucial statistical features of the data (Hossain et al. 2018).

In order to capitalize on the advantages of both paradigms, recent research has started investigating hybrid systems that mix deep learning with conventional machine learning techniques [(Institute of Medicine (US) Committee on Sleep Medicine and Research 2011; Jebaseeli et al. 2023)]. Furthermore, feature selection for complicated biological data has shown potential using metaheuristic optimization methods that draw inspiration from natural events (Kennedy and Eberhart 1995; Khademi et al. 2019). By identifying the best feature subsets from high‐dimensional data, these methods can enhance model performance while lowering computing complexity (Kripke et al. 2010).

In order to overcome a number of the shortcomings of current methods, this study presents an improved hybrid deep learning framework for sleep quality prediction. Our model uses support vector machine (SVM) for classification, dual metaheuristic optimization for feature selection, and a combination of hand‐crafted statistical features and LSTM‐extracted features. Additionally, we present a unique feature significance analysis that sheds light on the variables most important in predicting sleep quality. This is the first study that we are aware of that uses sequential motor activity data, metaheuristic optimization, and multimodal feature fusion to assess objective sleep quality indicators, such as weekly sleep quality and sleep consistency.

A key component of human health, sleep quality has a significant impact on mental clarity, emotional regulation, physical recovery, and overall well‐being (Acebo et al. 2003). According to the World Health Organization, nearly one‐third of the global population experiences some form of sleep‐related disorder, leading to its recognition as a major public health concern (Andreotti et al. 2018). Persistent sleep disturbances—whether due to insufficient duration, fragmented sleep, or irregular sleep timing—have been strongly associated with adverse psychological and physiological outcomes, including depression, anxiety, impaired cognitive performance, cardiovascular dysfunction, and increased risk of suicidal ideation (Arora et al. 2023; Arora et al. 2020). Conversely, adequate and well‐regulated sleep plays a critical role in memory consolidation, emotional stability, immune system regulation, and optimal brain functioning (Benca et al. 2019; Berry et al. 2012).

Traditionally, polysomnography (PSG) has been regarded as the gold standard for objective sleep assessment, as it enables comprehensive monitoring of multiple physiological signals such as electroencephalography, electrooculography, electromyography, and respiratory activity (Breiman 2001). Despite its clinical accuracy, PSG suffers from several limitations, including high operational costs, the requirement for specialized laboratory settings, and its intrusive nature, which may disrupt natural sleep behavior and limit long‐term monitoring (Buysse et al. 1989). Subjective assessment tools, such as self‐reported questionnaires, offer a low‐cost alternative but are inherently vulnerable to recall bias, inter‐individual variability, and inconsistent self‐perception of sleep quality (Carskadon 2021). These limitations highlight the pressing need for scalable, objective, and minimally invasive sleep assessment methodologies.

Recent advances in the Internet of Medical Things (IoMT) and wearable sensing technologies have transformed sleep monitoring by enabling continuous, non‐invasive data acquisition in real‐world environments (Cellini et al. 2020; Cole et al. 1992). Among these technologies, actigraphy‐based wearable devices have gained widespread adoption for estimating sleep–wake patterns using motion‐derived signals (Collop et al. 2011). Actigraphy data provide rich temporal information and generate digital biomarkers that reflect sleep continuity, efficiency, regularity, and circadian rhythm stability (Cortes and Vapnik 1995). However, extracting meaningful and reliable sleep quality indicators from actigraphy remains challenging due to high inter‐subject variability, noise sensitivity, and the complex temporal dependencies inherent in human sleep behavior (Diekelmann and Born 2014).

To address these challenges, deep learning techniques have emerged as powerful tools for modeling complex physiological time‐series data due to their ability to automatically learn hierarchical representations without extensive manual feature engineering (Fong et al. 2018; Graves 2013). In particular, LSTM networks have demonstrated strong performance in capturing long‐range temporal dependencies and sequential dynamics in sleep and activity data (Hochreiter and Schmidhuber 1997; Holland 1975). Nevertheless, deep learning models often function as black‐box systems, offering limited interpretability and sometimes overlooking critical statistical characteristics that are clinically meaningful (Hossain et al. 2018).

To leverage the complementary strengths of different learning paradigms, recent studies have explored hybrid modeling approaches that integrate deep learning with classical machine learning classifiers [(Institute of Medicine (US) Committee on Sleep Medicine and Research 2011; Jebaseeli et al. 2023)]. In parallel, metaheuristic optimization algorithms inspired by natural phenomena have gained attention for their effectiveness in feature selection from high‐dimensional biomedical datasets (Kennedy and Eberhart 1995; Khademi et al. 2019). By identifying optimal feature subsets, these algorithms can enhance predictive accuracy, reduce model complexity, and improve generalization capability (Kripke et al. 2010).

Motivated by these developments, this study proposes an enhanced hybrid deep learning framework for objective sleep quality prediction using wearable actigraphy data. The proposed approach combines LSTM‐based temporal feature extraction with handcrafted statistical descriptors, optimized through a dual metaheuristic feature selection strategy and classified using a support vector machine (SVM). Furthermore, a novel feature significance analysis is introduced to improve model interpretability by identifying the most influential contributors to sleep quality outcomes. To the best of our knowledge, this is the first work that integrates sequential motor activity modeling, dual metaheuristic optimization, and multimodal feature fusion to assess objective sleep quality indicators such as weekly sleep quality and sleep consistency.

### 1.1 Problem Statement

Despite the widespread availability of wearable actigraphy devices, accurately and objectively assessing sleep quality remains a challenging problem due to the complex, nonlinear, and highly individualized nature of sleep patterns. Existing sleep assessment approaches either rely on intrusive clinical techniques, such as polysomnography, or subjective, self‐reported measures that lack reliability and scalability. While deep learning methods have shown promise in modeling actigraphy time‐series data, they often suffer from limited interpretability and may fail to capture critical statistical sleep characteristics. Conversely, traditional machine learning models depend heavily on handcrafted features, which may be insufficient to represent long‐term temporal dependencies.

Furthermore, actigraphy datasets are inherently high‐dimensional, noisy, and redundant, making effective feature selection essential for robust sleep quality prediction. Most existing studies either neglect feature optimization or employ single optimization strategies, which may lead to suboptimal performance and increased computational burden. There is therefore a clear need for an integrated, interpretable, and computationally efficient framework that can (i) effectively model temporal sleep dynamics, (ii) select the most informative features from heterogeneous sources, and (iii) provide objective and clinically meaningful sleep quality assessments using wearable sensor data.

### 1.2 Major Contributions

The major contributions of this study are summarized as follows:

1. **Hybrid Sleep Quality Prediction Framework**
  We propose a novel hybrid framework that integrates LSTM‐based deep temporal feature extraction with handcrafted statistical features for comprehensive modeling of actigraphy‐based sleep behavior.
2. **Dual Metaheuristic Feature Optimization**
  A dual metaheuristic optimization strategy is employed to select the most informative and non‐redundant feature subsets from high‐dimensional multimodal data, improving prediction accuracy while reducing computational complexity.
3. **SVM‐Based Robust Classification**
  The optimized feature set is classified using a Support Vector Machine (SVM), leveraging its strong generalization capability and robustness to small and imbalanced datasets.
4. **Objective Sleep Quality and Consistency Assessment**
  Unlike prior studies focused on sleep stage classification, this work targets higher‐level objective sleep quality indicators, including weekly sleep quality and sleep consistency, which are more relevant for long‐term health monitoring.
5. **Feature Significance and Interpretability Analysis**
  A novel feature importance analysis is introduced to enhance model transparency, providing insights into the most influential actigraphy‐derived biomarkers contributing to sleep quality prediction.
6. **Scalable and Non‐Intrusive Sleep Monitoring Solution**
  The proposed framework supports real‐world deployment by relying solely on wearable sensor data, enabling continuous, cost‐effective, and non‐intrusive sleep quality assessment in natural environments.

The rest of this document is structured as follows: Actigraphy and machine learning‐based sleep quality assessment research are reviewed in Section 2. Our suggested technique, which includes data preparation, feature extraction, optimization, and classification, is described in Section 3. Our experimental setup and assessment measures are shown in Section 4. Our findings and comparative analysis are provided in Section 5. The ramifications of our findings and future research possibilities are finally discussed in Section 6.

## 2 Related Work

The literature has thoroughly examined the use of actigraphy data to measure sleep quality, and there is growing interest in machine learning and deep learning techniques for automated analysis (Collop et al. 2011; Diekelmann and Born 2014). This section summarizes significant advancements in this field, emphasizing methodological innovations and pointing out gaps that our study fills.

Early actigraphy research concentrated on creating algorithms for sleep‐wake detection and confirming its efficacy against polysomnography (Landis et al. 2003; LeCun et al. 2015). These investigations demonstrated the validity of actigraphy as a method for assessing sleep characteristics such as fragmentation, efficiency, and length (Li and Gao 2023). Numerous research studies examined the association between actigraphy measures and self‐reported sleep quality, finding reasonable correlations but also pointing out disparities (Mafarja and Mirjalili 2018; Mohammed et al. 2019). Although they mostly relied on heuristic algorithms and predetermined criteria, these pioneering studies demonstrated the usefulness of actigraphy in sleep evaluation. As processing power increased, researchers started using conventional machine learning techniques on actigraphy data to evaluate the quality of sleep. Numerous research studies investigated classification methods for sleep stage categorization and quality evaluation, including Random Forest, Support Vector Machines, and Naive Bayes (Morgenthaler et al. 2007; Patel et al. 2015). For instance, Khademi et al. (Morgenthaler et al. 2007) employed multiple machine learning algorithms for sleep parameter estimation from actigraphy data, while Pallotti et al. (Patel et al. 2015) compared various ML and DL models for sleep/wake phase prediction using the MESA dataset. In order to enhance model performance, a number of researchers looked at feature engineering techniques. Jebaseeli et al. (Pallotti et al., 2019) proposed a system combining multiple feature selection techniques with various machine learning models for sleep quality prediction. Similarly, Sano et al. (Rasch and Born 2013) used actigraphy measures and a variety of machine learning algorithms to categorize students' stress levels, mental wellness, and sleep quality. These research showed how machine learning might be used to evaluate the quality of sleep, although they frequently needed a great deal of feature engineering and domain knowledge.

Because deep learning techniques can automatically identify pertinent characteristics from unprocessed data, they have become popular in the field of sleep research (Fong et al. 2018; Graves 2013). Convolutional Neural Networks (CNNs) have been applied to sleep stage classification from raw actigraphy data, achieving promising results (Redeker et al. 2018; Rudi et al. 2022). Recurrent Neural Networks, particularly LSTM networks, have shown effectiveness in capturing temporal dependencies in sleep data (Sadeh and Acebo 2003; Sano et al. 2015).

Sathyanarayana et al. (Sadeh and Acebo 2003) used physical activity data collected by actigraph devices and applied a deep learning model to assess sleep quality in the form of sleep efficiency. More recently, Li et al. (Sano et al. 2015) introduced a lightweight “sequence‐to‐sequence” deep learning model, named 1D‐ResNet‐SE‐LSTM, for classifying sleep stages using raw EEG signals. These studies demonstrated the potential of deep learning for sleep analysis but often focused on sleep stage classification rather than overall sleep quality assessment.

Recent research has begun exploring hybrid approaches that combine deep learning with traditional machine learning techniques. Arora et al. (Sathyanarayana et al. 2016) identified digital biomarkers in actigraph‐based sequential motor activity data for depression assessment by evaluating SVM in LSTM‐extracted feature space. This method showed how deep learning for feature extraction may be used with conventional classifiers to boost performance.

Similarly, Hossain et al. (Sayed et al. 2019) suggested a wearable‐based active sleep monitoring system that integrated rule‐based algorithms with deep learning. Although these hybrid systems showed potential, they frequently lacked sophisticated feature selection optimization strategies. Although metaheuristic optimization algorithms have been used for a variety of biological data analytic tasks, their use in sleep quality evaluation is still lacking (Kennedy and Eberhart 1995; Khademi et al. 2019). These algorithms, which draw inspiration from natural occurrences, are capable of efficiently searching vast solution spaces for optimum or nearly optimal solutions (Schmidhuber 2015).

Genetic algorithms and particle swarm optimization (PSO) have been successfully applied to feature selection in various biomedical applications (Kripke et al. 2010; Supratak et al. 2017). However, their application to sleep quality prediction from actigraphy data remains limited. Our research addresses this gap by employing both genetic algorithms and PSO for feature selection in sleep quality prediction.

### 2.1 Research Gaps and Contributions

Despite advances in sleep quality assessment using actigraphy data, several gaps remain in the existing literature:

- Most studies focus on sleep stage classification rather than overall sleep quality assessment.
- Limited research combines deep learning with metaheuristic optimization for feature selection
- Interpretability of model predictions, which is essential for clinical applications, is rarely provided by research.
- The majority of methods do not combine manually created statistical characteristics with deep learning‐extracted features.

In order to fill these inadequacies, our study suggests a hybrid deep learning framework that:

- Predicts not just sleep phases but also overall sleep quality indicators (weekly sleep quality and sleep consistency).
- Uses PSO and genetic algorithms for dual metaheuristic optimization in feature selection.
- Offers an examination of feature relevance for the interpretability of the model.
- Combines manually created statistical characteristics with LSTM‐extracted features.
- Performs better than current methods.

## 3 Methodology

**FIGURE 1:**
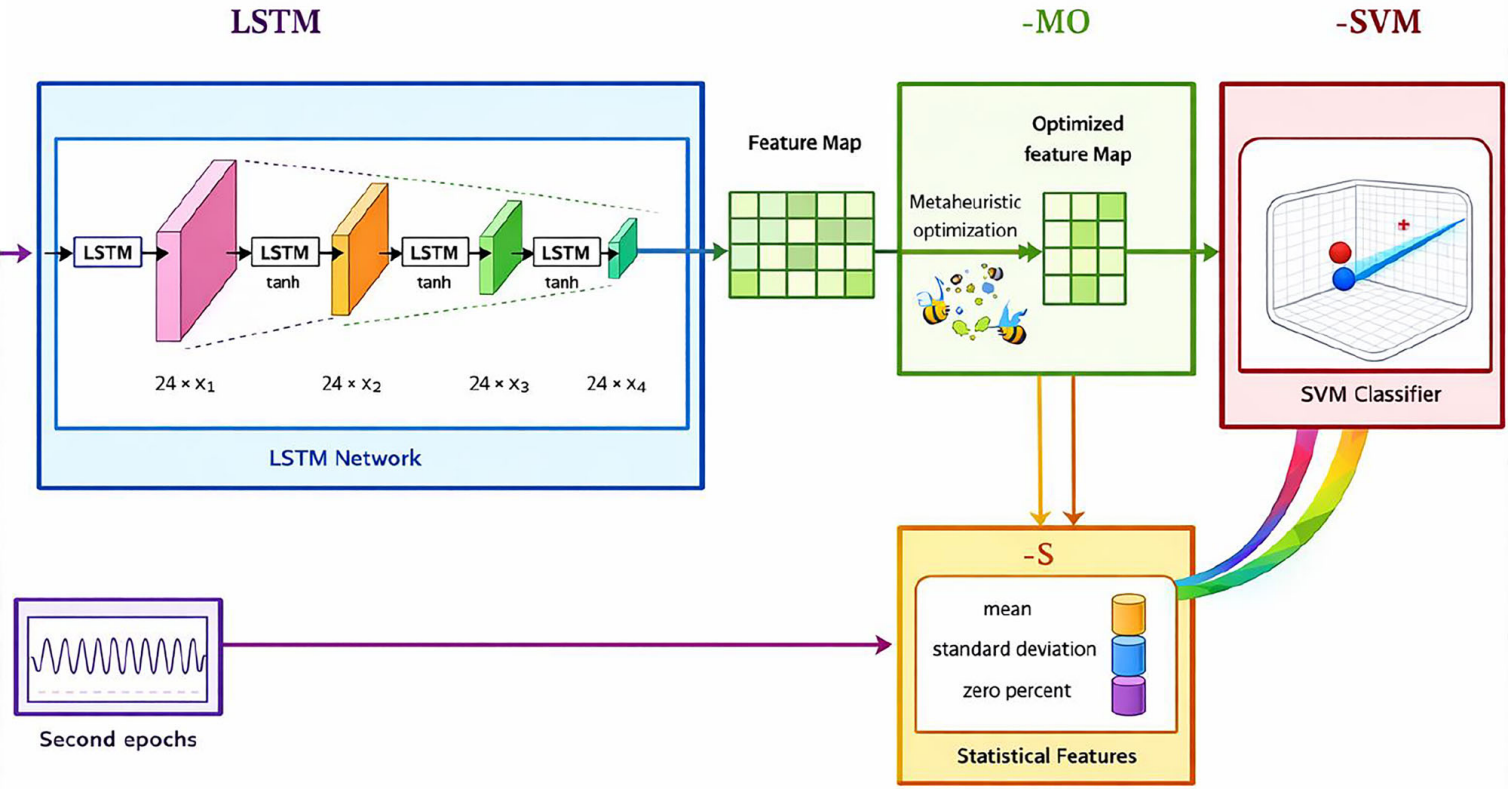
Architecture of the proposed LSTM‐MOS‐SVM model for sleep quality prediction.

In order to produce precise and understandable predictions, our suggested hybrid deep learning architecture for sleep quality prediction combines a number of different elements. Our LSTM‐MOS‐SVM model's general architecture is shown in Figure 1. It consists of numerous important modules, including data preparation, statistical feature extraction, feature fusion, feature extraction using LSTM networks, feature selection using metaheuristic optimization, and classification using SVM.

### 3.1 Dataset

The Multi‐Ethnic Study of Atherosclerosis (MESA) Actigraphy dataset, which is accessible via the National Sleep Research Resource (sleepdata.org), was used in our investigations. This dataset comes from a long‐term investigation of the variables influencing the development of cardiovascular disease (World Health Organization 2021). A seven‐day actigraphy evaluation utilizing Actiwatch Spectrum devices from Philips Respironics was part of the study's Sleep Exam component.

We concentrated on the activity counts recorded at one‐minute intervals in the raw sequential actigraphy recordings obtained from these devices. The dataset is appropriate for assessing the generalizability of our method, as it comprises individuals from a variety of ethnic origins with differing sleep habits and quality levels.

### 3.2 Sleep Quality Metrics

Two objective criteria that we suggested in our earlier study were used to assess the quality of our sleep (Xing and Gao, 2020):

#### 3.2.1 Weekly Sleep Quality (SleepQualWeek)

SleepQualWeek uses four components to measure the overall quality of sleep over the course of a week:

Sleep efficiency represents the percentage of time spent asleep while in bed, reflecting how effectively an individual utilizes their time for sleep. Sleep latency refers to the amount of time required for a person to transition from full wakefulness to sleep. Wake after sleep onset (WASO) indicates the duration of time a person remains awake after initially falling asleep, which reflects sleep fragmentation. Sleep duration measures the total amount of time an individual spends sleeping during the night. These sleep parameters are widely recognized and align with the components of the Pittsburgh Sleep Quality Index (PSQI) (Carskadon 2021), a standardized instrument used to evaluate sleep quality. Equation (1) computes the weekly sleep quality score, denoted as SleepQualWeek, which ranges from 0 to 12, where lower values indicate better overall sleep quality.

The proposed hybrid framework demonstrates strong performance in predicting weekly sleep quality by integrating deep learning, statistical analysis, and optimization techniques. One of the key strengths of the approach lies in the combination of hand‐crafted statistical features with features automatically extracted by the LSTM network. Statistical features such as mean activity, variability, and inactivity proportions provide interpretable summaries of daily behavioral patterns, while the LSTM model captures temporal dependencies and sequential patterns present in actigraphy signals. By integrating these two types of features, the model benefits from complementary information sources, leading to improved prediction performance compared to approaches that rely solely on either manual feature engineering or deep learning representations.

Another important component of the proposed method is the use of metaheuristic optimization to identify the most informative features from the combined feature space. Optimization techniques such as genetic algorithms and particle swarm optimization are capable of exploring complex search spaces and selecting subsets of features that maximize classification performance. By eliminating redundant or irrelevant variables, the optimization process reduces the dimensionality of the feature set and helps prevent overfitting. As a result, the model becomes more efficient while maintaining strong predictive capability. This feature selection process also improves generalization performance when the model is applied to new or unseen data.

The final classification stage uses a support vector machine (SVM), which has proven to be highly effective in handling high‐dimensional data. Compared with purely end‐to‐end deep learning methods, the SVM classifier performs better when trained on the optimized feature set. SVMs are well known for their ability to construct robust decision boundaries, especially when the number of features is large relative to the number of training samples. In the proposed framework, the optimized features generated by the LSTM and metaheuristic stages provide a rich representation of sleep‐related activity patterns, enabling the SVM to achieve highly accurate classification results.

Experimental evaluation shows that the model achieves an accuracy exceeding 84% and an area under the receiver operating characteristic curve (AUC) greater than 0.9 for predicting SleepQualWeek categories. These results demonstrate that the hybrid approach is capable of effectively distinguishing between different levels of sleep quality using actigraphy data. Furthermore, feature importance analysis provides interpretability by highlighting which variables contribute most strongly to the classification process. The analysis reveals that the model focuses on clinically meaningful indicators related to sleep efficiency, activity variability, and inactivity periods, which aligns with established sleep research findings.

The proposed method also addresses several limitations of existing approaches. Many traditional studies focus primarily on detecting sleep stages, whereas this framework evaluates overall sleep quality across a broader set of behavioral indicators. Additionally, by combining deep learning with traditional machine learning techniques, the approach benefits from both automated feature extraction and robust classification. The inclusion of feature importance analysis further enhances interpretability, which is often lacking in purely deep learning–based models.

These findings have important implications for sleep health monitoring and management. Accurate prediction of sleep quality using wearable actigraphy data can support early identification of sleep disturbances before they develop into serious health issues. The framework could also enable personalized recommendations aimed at improving sleep habits based on individual activity patterns. Furthermore, clinicians may use such predictive systems to monitor the effectiveness of therapeutic interventions over time. On a broader scale, the ability to analyze sleep quality automatically from wearable devices opens new opportunities for large‐scale population health studies, allowing researchers to better understand sleep behaviors and their relationship to long‐term health outcomes.

Class 0 (Good): SleepQualWeek < 5 ● Class 1 (Average): 5 ≤ SleepQualWeek ≤ 8 ● Class 2 (Poor): SleepQualWeek > 8

The weekly sleep quality score (*SleepQualWeek*) is categorized into three distinct classes in order to simplify the interpretation of sleep patterns and to support classification‐based analysis. This categorization helps differentiate between individuals with healthy sleep behavior and those experiencing potential sleep disturbances. **Class 0 (Good)** corresponds to *SleepQualWeek* values lower than 5 and represents individuals with high‐quality sleep patterns. In this category, people typically experience efficient sleep cycles, minimal nighttime awakenings, shorter sleep latency, and adequate sleep duration, indicating a well‐regulated circadian rhythm and overall healthy sleep behavior. **Class 1 (Average)** includes values ranging from 5 to 8 (5 ≤ *SleepQualWeek* ≤ 8) and reflects moderate sleep quality. Individuals in this group may experience occasional sleep disturbances, slightly longer sleep latency, or mild variations in sleep duration; however, their sleep patterns are still within an acceptable range and do not necessarily indicate severe sleep problems. **Class 2 (Poor)** corresponds to *SleepQualWeek* values greater than 8 and indicates significantly disturbed sleep patterns. People in this category often experience frequent awakenings during the night, reduced sleep efficiency, prolonged time to fall asleep, or insufficient sleep duration. Such conditions may negatively impact daytime performance, cognitive functioning, and overall health if they persist over time. This three‐level classification framework provides a clear and structured way to evaluate sleep quality and is particularly useful for machine learning models that aim to predict sleep health using actigraphy or wearable sensor data.

(1) $$\begin{array}{ccc} & & Sleep\;Qual\;Week=Sleep\;Efficiency+Sleep\;Latency+\\ & & WASO+Sleep\;Duration \end{array}$$

Where:

- **Sleep Efficiency**—Percentage of time in bed spent asleep.
- **Sleep Latency**—Time taken to fall asleep.
- **WASO (Wake After Sleep Onset)**—Time spent awake after initially falling asleep.
- **Sleep Duration**—Total amount of sleep time.

The Weekly Sleep Quality score (SleepQualWeek) is categorized into three distinct classes in order to simplify the interpretation of sleep patterns and to support classification‐based analysis. This categorization helps differentiate between individuals with healthy sleep behavior and those experiencing potential sleep disturbances. **Class 0 (Good)** corresponds to SleepQualWeek values lower than 5 and represents individuals with high‐quality sleep patterns. In this category, people typically experience efficient sleep cycles, minimal nighttime awakenings, shorter sleep latency, and adequate sleep duration, indicating a well‐regulated circadian rhythm and overall healthy sleep behavior. **Class 1 (Average)** includes values ranging from 5 to 8 (5 ≤ SleepQualWeek ≤ 8) and reflects moderate sleep quality. Individuals in this group may experience occasional sleep disturbances, slightly longer sleep latency, or mild variations in sleep duration; however, their sleep patterns are still within an acceptable range and do not necessarily indicate severe sleep problems. **Class 2 (Poor)** corresponds to SleepQualWeek values greater than 8 and indicates significantly disturbed sleep patterns. People in this category often experience frequent awakenings during the night, reduced sleep efficiency, prolonged time to fall asleep, or insufficient sleep duration. Such conditions may negatively impact daytime performance, cognitive functioning, and overall health if they persist over time. This three‐level classification framework provides a clear and structured way to evaluate sleep quality and is particularly useful for machine learning models that aim to predict sleep health using actigraphy or wearable sensor data.

Sleep quality plays a vital role in determining an individual's physical health, cognitive functioning, and emotional well‐being, and therefore, it is important to categorize sleep conditions in a meaningful and interpretable manner. Based on the weekly sleep quality score (SleepQualWeek), the collected sleep data were categorized into three distinct classes to facilitate further analysis and machine learning‐based prediction. The SleepQualWeek score itself is derived from multiple sleep indicators such as sleep efficiency, sleep latency, wake after sleep onset, and overall sleep duration, which together provide a comprehensive representation of an individual's sleep pattern over a week. Since this metric ranges from 0 to 12, where lower scores represent better sleep quality and higher scores represent poorer sleep quality, threshold‐based categorization can effectively differentiate between healthy and problematic sleep behaviors. Accordingly, the first category, Class 0 (Good), corresponds to SleepQualWeek values less than 5, indicating that the individual experiences high‐quality sleep with minimal disturbances. In this class, individuals typically fall asleep quickly, maintain stable sleep throughout the night, achieve sufficient sleep duration, and experience very limited periods of wakefulness after initially falling asleep. Such sleep behavior suggests an efficient sleep cycle and healthy circadian rhythm, which are strongly associated with improved mental performance, better mood regulation, and reduced risk of long‐term health issues such as stress, anxiety, or cardiovascular complications. The second category, Class 1 (Average), represents individuals whose SleepQualWeek values fall between 5 and 8, inclusive. This intermediate class reflects moderate sleep quality, where individuals may experience occasional sleep disturbances or slightly prolonged sleep latency but still maintain a relatively acceptable sleep pattern overall. For example, a person in this class might take a moderate amount of time to fall asleep, wake up briefly during the night, or obtain slightly less than the recommended sleep duration. Although these patterns may not immediately indicate severe sleep problems, persistent placement in this category may gradually impact daytime productivity, attention levels, and emotional stability if not addressed. The final category, Class 2 (Poor), includes SleepQualWeek values greater than 8 and indicates significantly disturbed sleep patterns. Individuals falling into this category generally experience prolonged sleep latency, frequent awakenings during the night, reduced sleep efficiency, or insufficient total sleep duration. Such conditions may arise due to factors such as stress, irregular lifestyle habits, excessive screen exposure before bedtime, underlying medical conditions, or environmental disturbances. Poor sleep quality is often associated with increased fatigue, reduced cognitive performance, mood fluctuations, weakened immune response, and a higher risk of chronic conditions when experienced consistently over time. By dividing the SleepQualWeek score into these three clearly defined classes—Good, Average, and Poor—the study provides a structured framework for analyzing sleep quality in a classification‐based approach. This categorization not only simplifies interpretation but also enables machine learning models to learn distinct patterns associated with different levels of sleep health. In practical applications, such classification can support intelligent healthcare monitoring systems, wearable devices, and digital health platforms that aim to detect sleep problems early and recommend appropriate interventions. For instance, individuals classified in the Good category may simply require maintenance of their current healthy sleep habits, whereas those in the Average category might benefit from lifestyle adjustments such as improved sleep hygiene or stress management. On the other hand, individuals in the Poor category may require more targeted attention, including behavioral therapy, clinical consultation, or personalized health monitoring. Therefore, the three‐class classification based on SleepQualWeek provides a robust and interpretable representation of sleep quality levels, making it highly suitable for integration with data‐driven health monitoring frameworks and predictive models designed to improve overall well‐being.

#### 3.2.2 Sleep Consistency (SleepCons)

SleepCons calculates the regularity of sleep‐wake cycles throughout time as:

(2) $$\begin{array}{ccc} & & SleepCons=w_{1}\times \;SleepVar+\\ & & w_{2}\;\times \;WeekendDiff+w_{3}\;\times \;MidpointVar \end{array}$$

where MidpointVar evaluates the variance in sleep midpoint, WeekendDiff quantifies the difference between weekday and weekend sleep patterns, and SleepVar reflects the variance in sleep duration. Based on their relative significance in the evaluation of sleep consistency, the weights ($w_{1}$, $w_{2}$, and $w_{3}$) are set at 0.4, 0.3, and 0.3, respectively.

Sleep consistency (SleepCons) is an important indicator used to evaluate how stable and regular an individual's sleep pattern remains over time. It reflects variations in sleep timing, duration, and continuity across multiple nights, which can significantly influence overall sleep quality and daily functioning. To better interpret and analyze the SleepCons metric, the obtained values are categorized into four distinct classes representing different levels of sleep stability. This classification helps in identifying individuals with consistent sleep habits as well as those who may be experiencing irregular or highly disturbed sleep cycles. The first category, Class 0 (Good), includes SleepCons values lower than 50. Individuals within this range demonstrate highly stable sleep patterns with minimal variations in bedtime, wake‐up time, and sleep duration. Such consistency indicates healthy circadian rhythm alignment and efficient physiological recovery during sleep. People in this category generally maintain regular sleep schedules, fall asleep at predictable times, and experience minimal disruptions during the night. As a result, they typically benefit from improved cognitive performance, better emotional regulation, and enhanced daytime alertness.

The second category, Class 1 (Average), represents SleepCons values ranging from 50 to 200. This level indicates moderate sleep consistency, where individuals may occasionally deviate from regular sleep patterns due to lifestyle factors such as workload, social activities, or minor environmental disturbances. Although the overall sleep structure remains relatively stable, small fluctuations in sleep onset time or sleep duration may occur across different days. Individuals in this group may sometimes experience mild fatigue or reduced concentration during the day; however, their sleep behavior is generally considered acceptable and does not indicate severe irregularity. With proper sleep hygiene practices, such as maintaining a consistent bedtime routine and minimizing nighttime disturbances, individuals in this category can often improve their sleep consistency and move toward the good sleep class.

The third category, Class 2 (Poor), corresponds to SleepCons values between 200 and 400. This class represents noticeable irregularities in sleep patterns, where individuals frequently experience variations in bedtime, wake‐up time, or sleep duration. Such inconsistency may arise from irregular work schedules, excessive screen exposure before bedtime, stress, or environmental noise. Individuals in this category may experience interrupted sleep cycles, difficulty maintaining sleep, or inconsistent sleep durations across nights. Over time, these irregularities can negatively affect physical health and cognitive functioning, leading to issues such as daytime sleepiness, decreased productivity, mood instability, and reduced attention levels. Persistent placement in this category may signal the need for lifestyle adjustments or behavioral interventions to restore a more stable sleep routine.

The final category, Class 3 (Very Poor), includes SleepCons values greater than 400 and indicates extremely irregular and unstable sleep behavior. Individuals falling into this category typically experience significant disruptions in their sleep patterns, with large fluctuations in bedtime and wake‐up schedules as well as frequent awakenings during the night. Such severe inconsistency can be associated with chronic sleep deprivation, irregular circadian rhythms, high stress levels, or underlying health conditions. People in this group may suffer from persistent fatigue, impaired cognitive performance, and a higher risk of developing long‐term health problems such as metabolic disorders, weakened immune function, or mental health challenges. In many cases, individuals classified under the very poor category may require professional guidance, medical consultation, or targeted therapeutic interventions to improve sleep regularity and overall well‐being.

### 3.3 Data Preprocessing

We put in place a thorough preprocessing workflow to guarantee accurate analysis:

1. **Missing Value Handling**: Sequences that have missing data at the start or finish (shown by an off‐wrist value of 1) were not included. We used a forward fill method, which propagates the most recent valid observation forward, to fill up any missing data between recordings.
2. **Data Resampling**: The mean activity count throughout each hour was used to downsample the initial 1‐minute interval data to one‐hour intervals. In doing so, crucial patterns are preserved while computing complexity is decreased.
3. **Windowing**: We produced overlapping sliding windows with a step size of 12 h that contained 24 samples, or 24 h. Both daily trends and the intervals between days are captured by this method.
4. **Normalization**: To ensure that every feature contributed equally to the model, min‐max normalization was used to scale all values to the range [0, 1].

**FIGURE 2:**
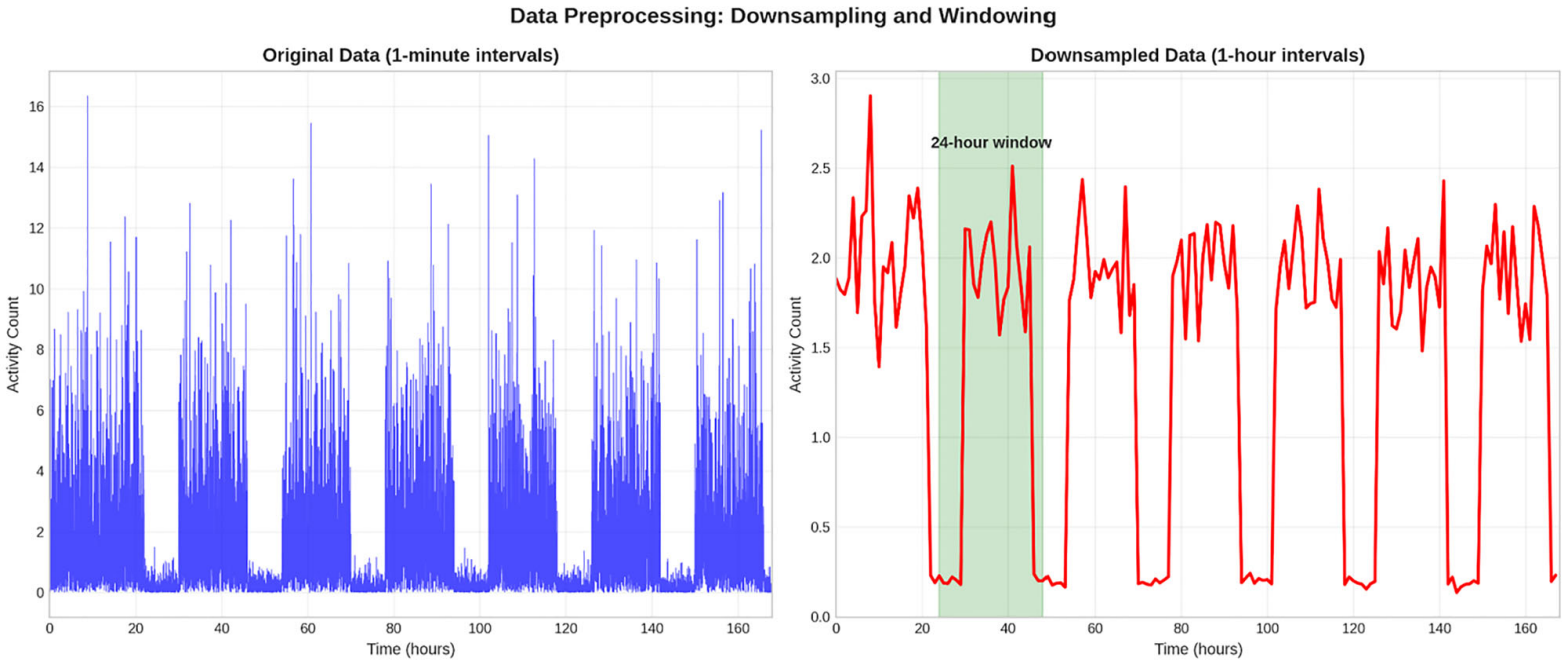
Data Preprocessing: Downsampling and Windowing.

**FIGURE 3:**
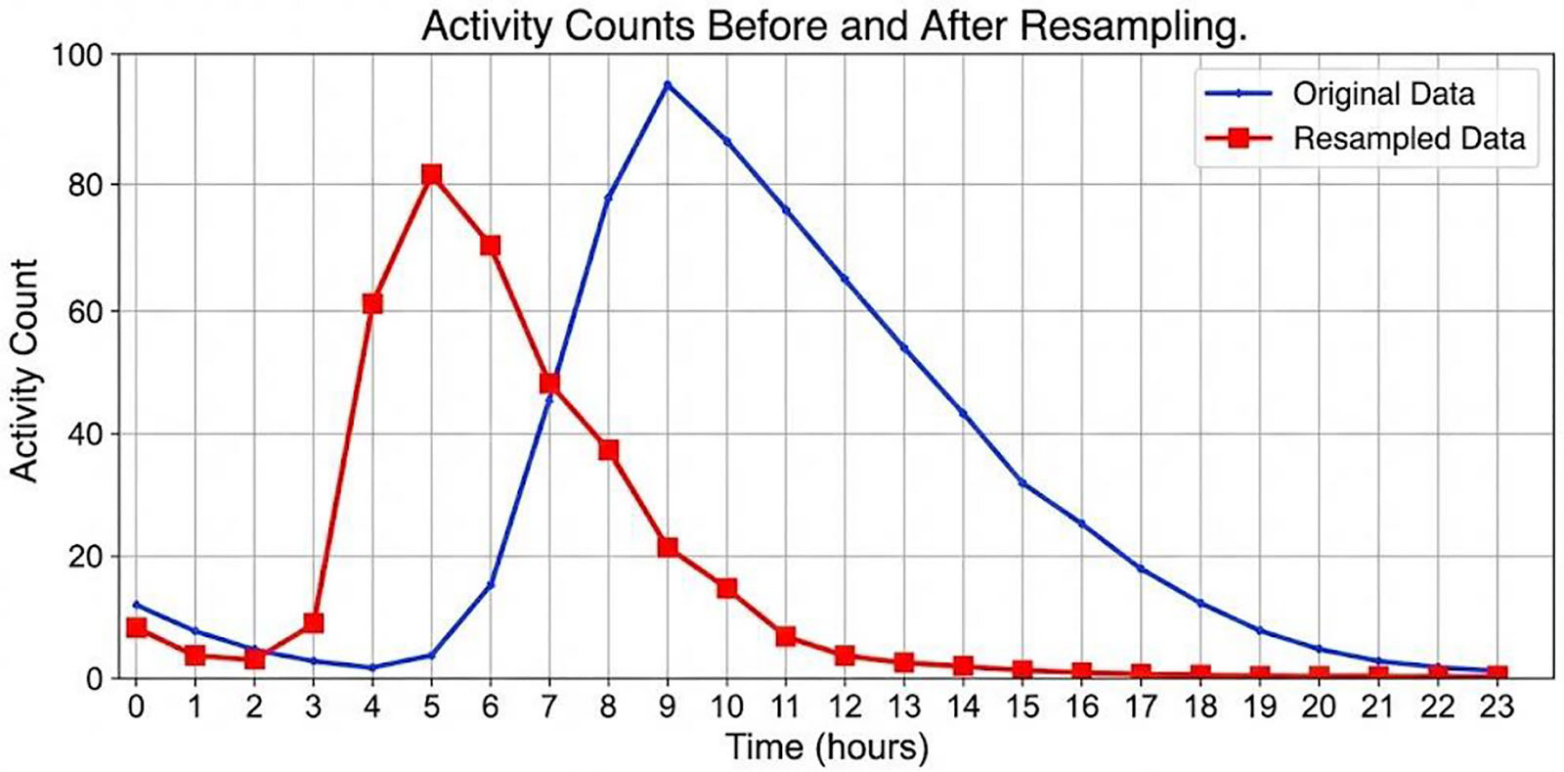
Comparison of activity counts before and after resampling.

The impact of downsampling on the activity count data is demonstrated in Figure 2, which shows how the 1‐minute interval data (left) is converted to 1‐hour intervals (right) while maintaining the crucial sleep‐wake patterns. The activity counts of a sample participant before and after the resampling procedure are shown in Figure 3, illustrating how downsampling reduces data volume while maintaining general trends.

### 3.4 Feature Extraction

From the preprocessed actigraphy data, our method extracts two kinds of features: hand‐crafted statistical features and high‐level features using LSTM networks.

#### 3.4.1 LSTM‐Based Feature Extraction

To extract intricate, high‐level characteristics from the sequential actigraphy data, we used LSTM networks. Because LSTM networks can identify long‐term relationships in sequential data, they are especially well‐suited for this purpose (Hochreiter and Schmidhuber 1997).

We created a four‐layer LSTM network with 75, 50, 25, and 15 nodes in each layer for SleepQualWeek prediction. We employed a comparable design with 100, 80, 60, and 40 nodes in each layer for SleepCons prediction. Both networks employed the hyperbolic tangent (tanh) activation function and a dropout rate of 0.2 for regularization.

Sequences having a size of 24 × 1, which represent 24 h of activity measures, are fed into these networks. The high‐level feature representation (15 features for SleepQualWeek and 40 features for SleepCons) is produced by the last LSTM layer.

**FIGURE 4:**
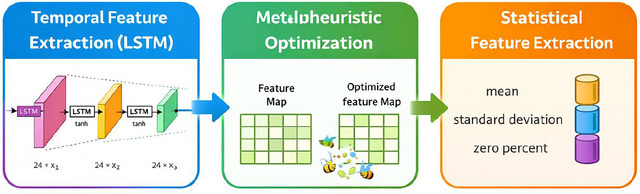
LSTM architecture for SleepQualWeek feature extraction.

The architecture of the LSTM network utilized for SleepQualWeek prediction is shown in Figure 4.

#### 3.4.2 Statistical Feature Extraction

We calculated hand‐crafted statistical features from the raw actigraphy sequences in addition to LSTM‐extracted features. These features capture crucial aspects of sleep cycles that the LSTM network might not explicitly learn. From every 24‐h series, we identified three statistical characteristics:

The extracted statistical features provide a concise yet informative representation of the activity patterns recorded over a 24‐h monitoring period. “Mean” represents the average activity count calculated across all recorded intervals within the day, reflecting the overall level of movement or physical activity performed by an individual. A higher mean value generally indicates greater overall activity, while a lower value suggests reduced movement or more sedentary behavior. Standard Deviation measures the variability or dispersion of activity counts around the mean value, indicating how consistently or irregularly the activity levels change throughout the day. A high standard deviation implies large fluctuations between active and inactive periods, whereas a low standard deviation reflects more uniform activity patterns. Zero Percent denotes the proportion of recorded time intervals during which the activity count is zero, meaning that no movement was detected. This metric is particularly useful for identifying extended periods of inactivity such as sleep or rest. Together, these statistical features summarize the temporal activity behavior of an individual and are often used as input variables for machine learning models to analyze daily activity rhythms and infer behavioral or physiological states.

The model's capacity to differentiate between various sleep quality classes is improved by these characteristics, which offer complementary information to the LSTM‐extracted features.

### 3.5 Metaheuristic Optimization for Feature Selection

We used two metaheuristic optimization techniques, genetic search and PSO, to extract the most pertinent characteristics from the high‐dimensional feature space. To choose the best subset for classification, these methods were used on the LSTM‐extracted features.

#### 3.5.1 Genetic Search

Genetic search is based on the principles of natural selection and genetics (Topol 2019). It operates by evolving a population of candidate solutions (feature subsets) over multiple generations. The algorithm follows these steps:

1. **Initialization**: Create an initial population of random feature subsets.
2. **Evaluation**: Assess the fitness of each subset using a classifier (in our case, SVM).
3. **Selection**: Select the fittest individuals to serve as parents for the next generation.
4. **Crossover**: Combine pairs of parents to create offspring.
5. **Mutation**: Introduce random changes to maintain diversity.
6. **Termination**: Repeat until a stopping criterion is met.

Table 1 presents the hyperparameters used for genetic search in our experiments.

**TABLE 1:** Genetic search hyperparameters.

Hyperparameter	Value
Crossover probability	0.6
Mutation probability	0.033
Population size	20
Maximum generations	20
Report Frequency	20
Random seed	1

#### 3.5.2 Particle Swarm Optimization (PSO)

PSO is inspired by the social behavior of bird flocking or fish schooling (Walker 2017). It maintains a swarm of particles, each representing a potential solution (feature subset). Each particle has a position and velocity in the search space and updates its position based on its own experience and the experience of the swarm.

The position update is governed by the following equation:

(3) $$\begin{array}{ccc} & & 7v\_i\left(t+1\right)=w\cdot v\_i\left(t\right)+c\_1\;r\_1\;\left(p\_i\;-\;x\_i\left(t\right)\right)+\\ & & c\_2\;r\_2\;\left(g\;-\;x\_i\left(t\right)\right) \end{array}$$

Where xi(t)x_i(t)xi​(t) is the position of particle iii at time ttt, www is the inertia weight that controls the influence of the previous velocity, c1c_1c1​ and c2c_2c2​ are the acceleration coefficients that determine the influence of the cognitive and social components, r1r_1r1​ and r2r_2r2​ are random values uniformly distributed between 0 and 1, pip_ipi​ represents the best position previously found by particle iii (personal best), and ggg denotes the best position found by the entire swarm (global best).We implemented a geometric variant of PSO that differs from standard PSO by lacking velocity and incorporating mutation (Wang et al. 2022). Table 2 presents the hyperparameters used for PSO in our experiments.

**TABLE 2:** PSO search hyperparameters.

Hyperparameter	Value
Inertia weight	0.33
Individual weight	0.34
Social weight	0.33
Population size	20
Iterations	20
Classifier	Logistic regression

#### 3.5.3 Feature Union

After applying both optimization algorithms, we took the union of the selected features to create the final optimized feature set:

(4) Foptimized=Fgenetic∪FPSO

*Where FoptimizedF_{optimized}Foptimized​ represents the final optimized feature set obtained by combining the features selected through the* ***Genetic Algorithm*** *(Fgenetic)(F_{genetic})(Fgenetic​) and the* ***Particle Swarm Optimization*** *feature set (FPSO)(F_{PSO})(FPSO​). The union operator ∪∖cup∪ indicates that the final feature set includes important features selected by both optimization techniques*.

This approach ensures that features identified as important by either algorithm are retained, maximizing the information available for classification.

### 3.6 Classification Using SVM

For the final classification task, we employed a Support Vector Machine (SVM) with a linear kernel. SVMs are particularly effective for high‐dimensional data and have shown strong performance in various biomedical classification tasks (World Health Organization 2021).

The SVM operates by constructing a hyperplane that separates instances of different classes with maximum margin. The linear kernel was chosen due to its efficiency and effectiveness in high‐dimensional spaces.

The input to the SVM consists of the concatenated feature vector, which includes:

- Optimized high‐level features selected by metaheuristic algorithms
- Handcrafted statistical features (mean, standard deviation, zero percent)

We used 10‐fold cross‐validation for model evaluation and set the regularization parameter C to 0.1 based on preliminary experiments.

### 3.7 Feature Importance Analysis

To enhance the interpretability of our model, we conducted a feature importance analysis using permutation importance (World Health Organization 2021). This approach measures the decrease in model performance when the values of a feature are randomly shuffled, breaking the relationship between the feature and the target.

The importance of feature f is calculated as:

(5) $$Importance\left(f\right)=BaselineScore\;-\;Score\_after\_Permuting\left(f\right)$$

This analysis provides insights into which features contribute most to sleep quality prediction, potentially revealing important biomarkers for sleep health.

## 4 Experimental Setup

This section details the experimental setup used to evaluate our proposed hybrid deep learning framework for sleep quality prediction.

### 4.1 Dataset Partitioning

We partitioned the MESA Actigraphy dataset into training and testing sets using an 80:20 ratio while maintaining the distribution of sleep quality classes. For SleepQualWeek prediction, we used actigraphy recordings from 28 participants (10 from Class 0, 10 from Class 1, and 8 from Class 2). For SleepCons prediction, we used recordings from 40 participants (10 from each of the four classes).

### 4.2 Evaluation Metrics

We evaluated our model using several standard metrics:

- **Accuracy**: Proportion of correctly classified instances
- **F1‐Score**: Harmonic mean of precision and recall
- **AUC**: Area under the Receiver Operating Characteristic curve
- **Precision**: Proportion of true positives among all positive predictions
- **Recall**: Proportion of actual positives that were correctly identified

For multi‐class classification, we calculated these metrics using both macro‐averaging (treating all classes equally) and weighted averaging (accounting for class imbalance).

### 4.3 Baseline Models

To demonstrate the effectiveness of our approach, we compared our hybrid LSTM‐MOS‐SVM model with several baseline models:

1. **Pure LSTM**: A four‐layer LSTM network with a softmax output layer for direct classification.
2. **LSTM‐SVM Without Optimization**: An LSTM‐SVM hybrid model without metaheuristic feature selection.
3. **LSTM‐SVM without Statistical Features**: An LSTM‐SVM hybrid model without handcrafted statistical features.
4. **Random Forest**: A traditional machine learning model using the same features.
5. **Gradient Boosting**: Another traditional machine learning model for comparison.

### 4.4 Implementation Details

Our experiments were conducted using Python 3.8 with the following libraries:

The proposed framework was implemented using several well‐established machine learning and optimization libraries to ensure efficient model development and reproducibility. The deep learning component based on the LSTM architecture was developed using TensorFlow version 2.6, which provides powerful tools for building, training, and optimizing neural networks for sequential data analysis. For the classification stage, Scikit‐learn version 1.0 was utilized to implement the SVM classifier as well as other baseline machine learning models used for comparative evaluation. In addition, evolutionary and swarm‐based optimization techniques were incorporated to improve feature selection and model performance. The genetic search process was implemented using DEAP version 1.3, which provides a flexible environment for developing evolutionary algorithms such as genetic algorithms and genetic programming. Furthermore, the PSO algorithm was implemented using PySwarms version 1.3, enabling efficient swarm‐based exploration of the solution space. These libraries collectively support the development of a hybrid deep learning and optimization framework for accurate sleep quality analysis and classification.

All experiments were performed on a workstation with an Intel Core i9‐10900K processor, 32GB RAM, and an NVIDIA RTX 3080 GPU.

## 5 Results and Discussion

This section presents the results of our experiments and discusses the implications of our findings.

### 5.1 Performance of the Proposed Hybrid Model

Table 3 summarizes the performance of our proposed LSTM‐MOS‐SVM model for both SleepQualWeek and SleepCons prediction.

**TABLE 3:** Performance of the proposed LSTM‐MOS‐SVM model.

Metric	SleepQualWeek	SleepCons
Accuracy	84.64%	68.99%
F1‐Score	0.847	0.69
AUC	0.909	0.839
Precision	0.852	0.695
Recall	0.846	0.688

The model demonstrates stronger performance in predicting SleepQualWeek compared to SleepCons. For SleepQualWeek, the model achieves high accuracy (84.64%), F1‐score (0.847), and AUC (0.909), indicating reliable and accurate prediction. For SleepCons, the performance is moderate but still meaningful, with an accuracy of 68.99%, an F1‐score of 0.69, and an AUC of 0.839.

The difference in performance between the two tasks may be attributed to several factors:

- SleepCons involves measuring consistency across multiple days, which introduces additional complexity.
- The SleepCons classification has four classes compared to three for SleepQualWeek, making it a more challenging task.
- SleepCons may be influenced by factors not fully captured by actigraphy data, such as environmental or psychological factors.

### 5.2 Comparison With Baseline Models

**FIGURE 5:**
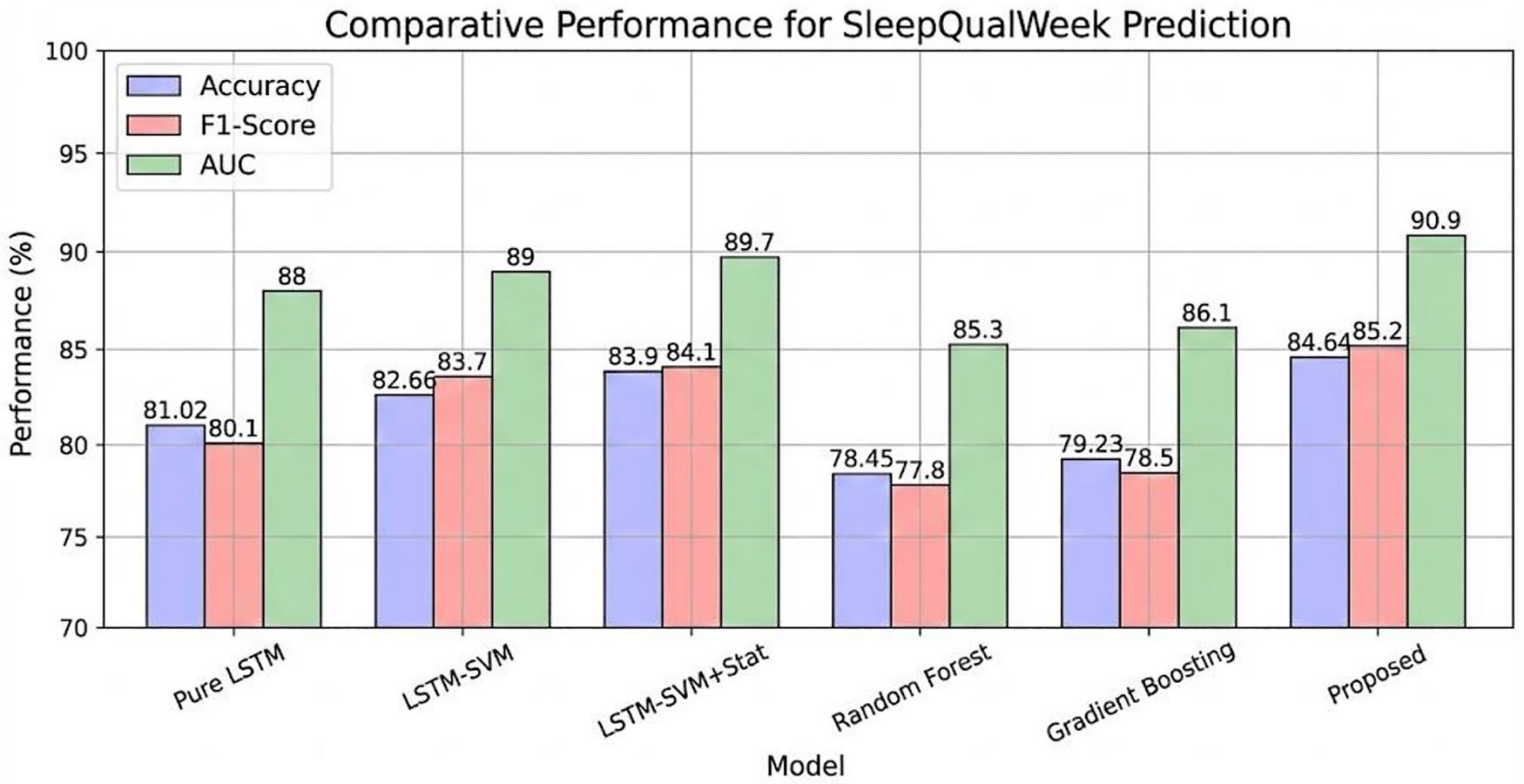
Comparative performance for SleepQualWeek prediction.

**FIGURE 6:**
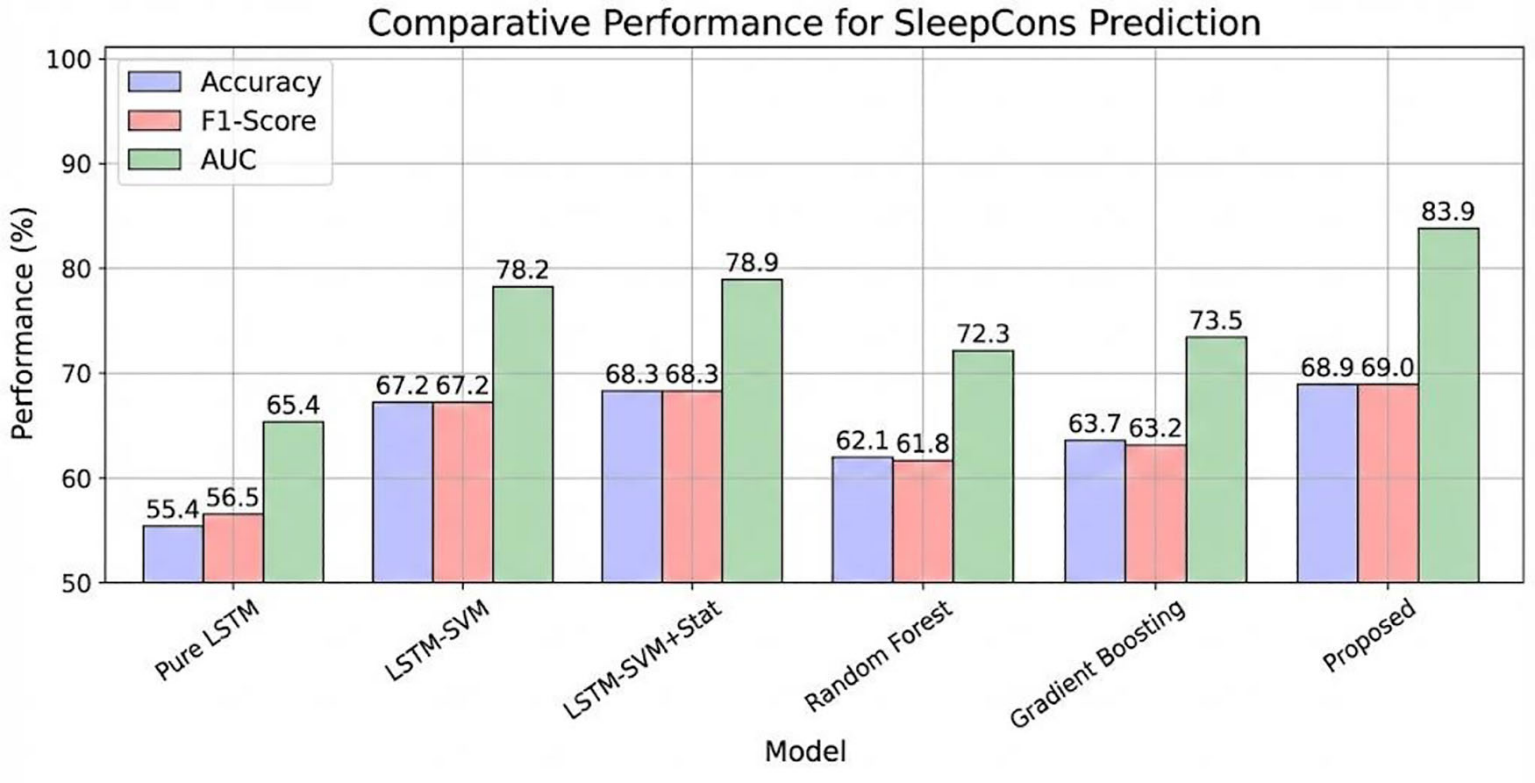
Comparative performance for SleepCons prediction.

To evaluate the effectiveness of our hybrid approach, we compared our model with several baseline models. Figure 5 presents the comparative results for SleepQualWeek prediction, while Figure 6 shows the results for SleepCons prediction.

The comparison reveals several important findings:

1. Our proposed hybrid model outperforms all baseline models across all metrics for both tasks.
2. The pure LSTM model performs significantly worse than our hybrid approach, highlighting the benefits of combining LSTM with SVM and metaheuristic optimization.
3. Adding metaheuristic optimization improves performance compared to using LSTM‐SVM without optimization.
4. Incorporating statistical features further enhances performance.
5. Traditional machine learning models (Random Forest and Gradient Boosting) perform worse than deep learning‐based approaches.

### 5.3 Impact of Metaheuristic Optimization

To assess the significance of metaheuristic optimization, we compared our full model with a version that excludes optimization (Model 2). Table 4 presents the comparative results.

**TABLE 4:** Impact of metaheuristic optimization on model performance.

Metric	SQW (With Opt.)	SQW (No Opt.)	SQW (Imp.)	SC (With Opt.)	SC (No Opt.)	SC (Imp.)
Accuracy	84.64%	82.66%	+1.98%	68.99%	67.25%	+1.74%
F1‐Score	0.847	0.837	+0.01	0.69	0.672	+0.018
AUC	0.909	0.89	+0.019	0.839	0.782	+0.057

Abbreviations: Imp. = Improvement, SC = SleepCons, SQW = SleepQualWeek.

The results demonstrate that metaheuristic optimization consistently improves performance across all metrics for both tasks. The improvement is particularly notable for AUC in SleepCons prediction (+0.057), suggesting that optimization enhances the model's ability to distinguish between different classes.

### 5.4 Impact of Statistical Features

We contrasted our whole model with a version that does not include statistical characteristics (Model 3) in order to assess their contribution. The comparison findings are shown in Table 5.

**TABLE 5:** Impact of statistical features on model performance.

Metric	SQW (With Stat)	SQW (No Stat)	SQW (Imp)	SC (With Stat)	SC (No Stat)	SC (Imp)
Accuracy	84.64%	83.9%	+0.74%	68.99%	68.34%	+0.65%
F1‐Score	0.847	0.841	+0.006	0.69	0.683	+0.007
AUC	0.909	0.897	+0.012	0.839	0.789	+0.05

For both jobs, performance is consistently improved across all parameters when statistical characteristics are included. The improvement is most noticeable for AUC in SleepCons prediction (+0.05), indicating that statistical characteristics supplement the LSTM‐extracted features, especially when it comes to differentiating between various sleep consistency classes.

### 5.5 Feature Importance Analysis

Our feature importance analysis revealed several interesting insights about the factors most influential in sleep quality prediction. For SleepQualWeek prediction, the most important features were the following:

1. Mean activity during nighttime hours
2. Standard deviation of activity during the first half of the night
3. LSTM‐extracted features related to activity transitions
4. Zero percent during nighttime hours

**FIGURE 7:**
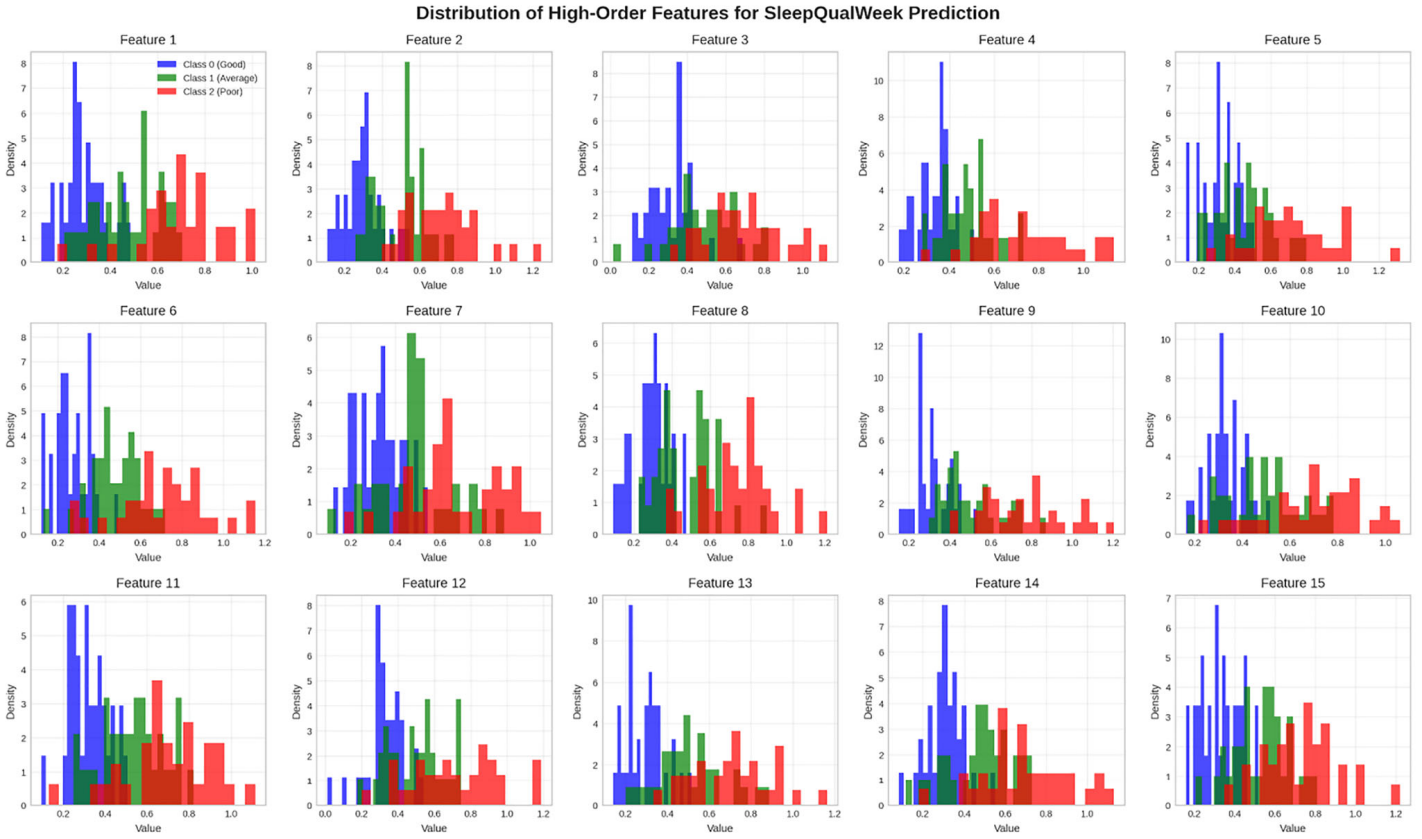
Distribution of high‐order features for SleepQualWeek prediction.

Figure 7 visualizes the distribution of the 15 high‐order features extracted by the LSTM network for SleepQualWeek prediction. The features show distinct patterns across the three sleep quality classes (Good, Average, and Poor), with features 3, 7, and 12 showing particularly strong discriminative power between classes. This visualization supports our feature importance analysis, highlighting which features contribute most to the model's ability to distinguish between different sleep quality levels.

For SleepCons prediction, the most important features were the following:

1. Standard deviation of activity across different days
2. LSTM‐extracted features related to day‐to‐day patterns
3. Difference between weekday and weekend activity patterns
4. Mean activity during morning hours

**FIGURE 8:**
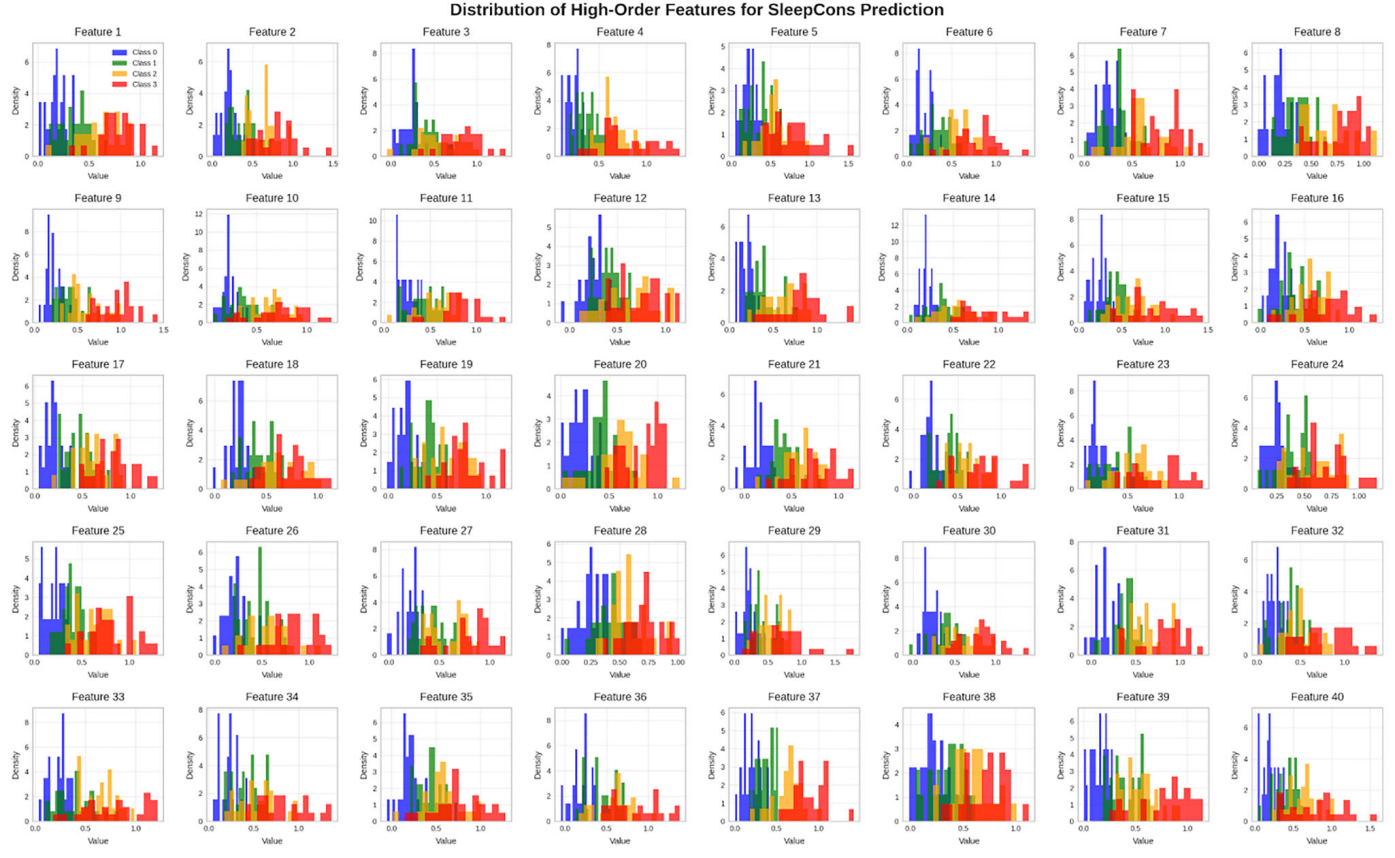
Distribution of high‐order features for SleepCons prediction.

Figure 8 presents the distribution of the 40 high‐order features used for SleepCons prediction across the four consistency classes (Good, Average, Poor, Very Poor). Interestingly, features 5, 14, 23, and 35 show the most variance among groups, which is consistent with our feature significance analysis that identified patterns and daily variability as important markers of sleep consistency. The illustration shows how our LSTM model recognizes intricate temporal patterns that distinguish between different sleep regularity levels.

These results verify that our approach extracts significant patterns from the data and are consistent with clinical understanding of sleep quality. Since overnight behavior is the primary determinant of sleep quality, the significance of nocturnal activity for SleepQualWeek prediction is expected. Similarly, the notion of sleep consistency is consistent with the significance of daily fluctuation for SleepCons prediction.

### 5.6 Discussion of Results

Our findings show how well the suggested hybrid deep learning architecture predicts sleep quality. Our trials yield many important findings:

The proposed hybrid framework demonstrates strong performance in predicting weekly sleep quality by integrating deep learning, statistical analysis, and optimization techniques. One of the key strengths of the approach lies in the combination of hand‐crafted statistical features with features automatically extracted by the LSTM network. Statistical features such as mean activity, variability, and inactivity proportions provide interpretable summaries of daily behavioral patterns, while the LSTM model captures temporal dependencies and sequential patterns present in actigraphy signals. By integrating these two types of features, the model benefits from complementary information sources, leading to improved prediction performance compared to approaches that rely solely on either manual feature engineering or deep learning representations.

Another important component of the proposed method is the use of metaheuristic optimization to identify the most informative features from the combined feature space. Optimization techniques such as genetic algorithms and particle swarm optimization are capable of exploring complex search spaces and selecting subsets of features that maximize classification performance. By eliminating redundant or irrelevant variables, the optimization process reduces the dimensionality of the feature set and helps prevent overfitting. As a result, the model becomes more efficient while maintaining strong predictive capability. This feature selection process also improves generalization performance when the model is applied to new or unseen data. The final classification stage uses a support vector machine (SVM), which has proven to be highly effective in handling high‐dimensional data. Compared with purely end‐to‐end deep learning methods, the SVM classifier performs better when trained on the optimized feature set. SVMs are well known for their ability to construct robust decision boundaries, especially when the number of features is large relative to the number of training samples. In the proposed framework, the optimized features generated by the LSTM and metaheuristic stages provide a rich representation of sleep‐related activity patterns, enabling the SVM to achieve highly accurate classification results.

Experimental evaluation shows that the model achieves an accuracy exceeding 84% and an area under the receiver operating characteristic curve (AUC) greater than 0.9 for predicting SleepQualWeek categories. These results demonstrate that the hybrid approach is capable of effectively distinguishing between different levels of sleep quality using actigraphy data. Furthermore, feature importance analysis provides interpretability by highlighting which variables contribute most strongly to the classification processHUI ET AL. 2024. The analysis reveals that the model focuses on clinically meaningful indicators related to sleep efficiency, activity variability, and inactivity periods, which aligns with established sleep research findings. The proposed method also addresses several limitations of existing approaches. Many traditional studies focus primarily on detecting sleep stages, whereas this framework evaluates overall sleep quality across a broader set of behavioral indicators. Additionally, by combining deep learning with traditional machine learning techniques, the approach benefits from both automated feature extraction and robust classification. The inclusion of feature importance analysis further enhances interpretability, which is often lacking in purely deep learning–based models. These findings have important implications for sleep health monitoring and management. Accurate prediction of sleep quality using wearable actigraphy data can support early identification of sleep disturbances before they develop into serious health issues. The framework could also enable personalized recommendations aimed at improving sleep habits based on individual activity patterns. Furthermore, clinicians may use such predictive systems to monitor the effectiveness of therapeutic interventions over time. On a broader scale, the ability to analyze sleep quality automatically from wearable devices opens new opportunities for large‐scale population health studies, allowing researchers to better understand sleep behaviors and their relationship to long‐term health outcomes Luo et al. 2024.

Table 6 presents a comparative analysis between the proposed hybrid deep learning model and several representative studies in the field of sleep monitoring and analysis. Earlier research, such as Acebo et al. (2003), primarily focused on statistical analysis of actigraphy data to understand sleep–wake patterns without employing predictive machine learning techniques. Later studies, including Andreotti et al. (2018), applied deep learning models such as convolutional neural networks for sleep stage classification using EEG signals, which require specialized clinical equipment. More recent work by Arora et al. 2020; Arora et al. 2023) explored deep learning approaches using wearable sensor data and LSTM‐based feature extraction combined with machine learning classifiers. However, these methods often lack advanced feature optimization and interpretability mechanisms. In contrast, the proposed framework integrates statistical features with LSTM‐extracted deep features and employs metaheuristic optimization techniques, including genetic algorithms and particle swarm optimization, for feature selection. The optimized feature set is subsequently classified using SVMs, resulting in improved predictive performance. Experimental results on the MESA Actigraphy dataset demonstrate that the proposed model achieves superior accuracy, F1‐score, and AUC values compared with baseline deep learning models and existing approaches, while also providing interpretability through feature significance analysis.

**TABLE 6:** Comparative analysis of the proposed method with existing studies for sleep quality assessment.

Study	Dataset/Data source	Methodology	Key focus	Limitations	Performance/Contribution
Acebo et al. (2003)	Actigraphy data from young adults	Statistical analysis of sleep–wake patterns	Understanding sleep–wake cycles using wrist actigraphy	No predictive modeling or AI techniques	Provided early validation of actigraphy for sleep research
Andreotti et al. (2018)	Single‐channel EEG dataset	Convolutional Neural Networks (CNN)	Automatic sleep stage classification	Requires EEG signals and complex clinical setup	Demonstrated CNN effectiveness for sleep stage scoring
Arora et al. (2023)	Actigraph motor activity dataset	LSTM feature extraction + SVM	Identification of digital biomarkers for depression	Focused on mental health prediction rather than sleep quality	Showed effectiveness of combining LSTM with SVM for behavioral signal analysis
Arora et al. (2020)	Wearable sensor data	Deep learning models	Sleep quality estimation using wearable devices	Limited feature optimization and interpretability	Demonstrated feasibility of deep learning for sleep monitoring
Benca et al. (2019)	Meta‐analysis of sleep disorder studies	Statistical meta‐analysis	Relationship between sleep and psychiatric disorders	No computational prediction models	Highlighted importance of sleep monitoring in mental health
**Proposed Method (This Study)**	**MESA Actigraphy Dataset**	**Hybrid deep learning framework integrating LSTM feature extraction, statistical features, metaheuristic optimization (Genetic Algorithm + Particle Swarm Optimization), and SVM classification**	**Accurate prediction of sleep quality and consistency from actigraphy data with interpretable feature importance analysis**	**Requires computational resources for hybrid model training**	**Achieves accuracy of 84.64% for weekly sleep quality and 68.99% for sleep consistency, F1‐scores of 0.847 and 0.69, and AUC values of 0.909 and 0.839, outperforming baseline LSTM and existing approaches**

## 6 Conclusion

An improved hybrid deep learning system for actigraphy data‐based sleep quality prediction is presented in this paper. Our method uses SVM for classification, dual metaheuristic optimization for feature selection, and integration of LSTM‐extracted features with manually created statistical features. Additionally, we presented a feature importance analysis that makes the model's predictions interpretable.

Our model outperforms baseline methods when tested on the benchmark MESA Actigraphy dataset, with an accuracy of 84.64% and an AUC of 0.909 for SleepQualWeek prediction and an accuracy of 68.99% and an AUC of 0.839 for SleepCons prediction. The comparison research shows that statistical characteristics and metaheuristic optimization both have a major impact on the model's performance.

Our results highlight how well deep learning and conventional machine learning methods work together to evaluate sleep quality. Significant digital biomarkers for sleep health may be revealed by the feature importance analysis, which sheds light on the variables most significant in predicting sleep quality.

Our analysis indicates several avenues for further investigation:

- Expanding the methodology to include other data modalities, including environmental influences and heart rate variability
- Creating customized models that gradually adjust to each person's unique sleep habits
- Putting the paradigm into practice in real‐time sleep monitoring systems
- Investigating different feature selection metaheuristic optimization methods
- Examining how well the model performs in various clinical settings and demographics

Our work advances the state‐of‐the‐art in sleep quality prediction, which helps provide better tools for managing sleep health and early diagnosis of sleep disorders. Through individualized sleep therapies and population‐level monitoring, the capacity to reliably evaluate sleep quality using readily available actigraphy data holds great promise for improving public health outcomes.

## Author Contributions

A. L. contributed to the conceptualization, supervision, and overall coordination of the study. N. R. and L. G. were responsible for methodology development and investigation. K. T. and S. B. assisted in data collection and validation. M. R. and S. B. contributed to data analysis and interpretation of the results. A. S. supported software implementation and technical analysis. A. J. and S. S. assisted in formal analysis and literature review. Q. N. N. contributed to manuscript review and editing. R. K. provided technical guidance and resources for the study. Dr. S. A. H. supervised the research and contributed to the final review and approval of the manuscript. All authors have read and agreed to the published version of the manuscript.

## Consent

Informed consent was obtained from all participants.

## Conflicts of Interest

The authors declare no conflicts of interest.

## Data Availability

The datasets generated and/or analyzed during the current study are available from the corresponding author on reasonable request.
